# Supplementary material for: Angiotensin-Converting Enzyme (ACE) gene polymorphism and arterial blood pressure among the Tawang Monpa of Eastern Himalayan Mountains: Is there a signature of natural selection?
Source: PLoS One. 2023 Sep 21;18(9):e0291810. doi: 10.1371/journal.pone.0291810 (PMC10513219; doi:10.1371/journal.pone.0291810)
Supplement: Attachment — Submitted filename: Ghosh_Response.docx [file pone.0291810.s002.docx]

## Dr. Emily Chenette,

Professor

Editor-in-Chief

PLOS ONE

Dear Prof. Chenette,

August 30, 2023

Re: MS#PONE-D-23-15795 entitled, “Angiotensin Converting Enzyme (ACE) gene polymorphism and arterial blood pressure among the Tawang Monpa of Eastern Himalayan Mountains: Is there a signature of natural selection?” by Sudipta Ghosh.

I am grateful to you and the reviewers for thoroughly reviewing this paper. I gratefully appreciate the reviewers’ time and efforts invested in critically reading this manuscript. I have carefully considered all the comments and I have provided a list of my responses, corrections, and detailed answers presented point-by-point in the order of the comments below.

I hope the revised version of this MS is now suitable for publication in PLOS ONE*.*

Sudipta Ghosh, PhD

Assistant Professor, Corresponding author

**Responses to the comments**

**Reviewer # 1 Comments:**

1) The number of individuals analysed in this study is relatively small, which limits its statistical power. I would suggest either increasing the sample size or including a thorough discussion about the limitations posed by the current sample size in the discussion section of the paper.

Author’s response: I agree with the reviewer. The sample size of the present study is not on the upper side of the limit. However, since it is not possible to increase the sample size at this point, I have given a thorough discussion about the sample size limitation in the discussion section of the manuscript, as follows:

*“......the sample size of the present study being on the lower side of robust analysis, it is important to conduct further study with larger sample size for better understanding of the scenario.”*(p. 13).

2) Adaptation to high altitudes is a multifactorial process and is not solely linked to the behaviour of the ACE genotypes. Hence to address this substantial biological question more comprehensively, I recommend integrating an analysis of other genes that play a significant role in high-altitude adaptation, such as EPAS1, EGLN1, PDE4D, HBB, HBA1, HBA2, VRGFA, and/or PPARGC1A

Author’s response: I thank the reviewer for pointing out this. While agreeing with the reviewer, I want to mention here that the focal theme of the paper is to understand the role of the ACE genotype in high-altitude adaptation among humans.

However, following the reviewer's suggestions, I will analyze the role of other important candidate genes and try to understand their roles among the Monpa in my following paper.

**Reviewer #2 Comments:**

1. Place the p<0.001 value symbol at each value that obtained it, in order to be consistent with the text “The genotype frequency of I/D heterozygote (0.48) was significantly higher (χ^2^=16.75; p<0.001) than the genotype frequencies of both I/I (0.26) and D/D (0.26) homozygotes (Table 2). The allele frequencies of the -I and -D allele were identical (0.50).”

Author’s response: I am thankful to the reviewer for this suggestion. Following this suggestion, I have inserted “p<0.001” in the appropriate places in the above sentence.

**Academic Editor’s Comments:**

1. **Methodology and Statistical Analysis**: Your manuscript contains an extensive statistical analysis of anthropometric, physiological, and genotypic characteristics. While the analysis is robust, the reviewers suggest that it could be helpful to provide a clearer explanation of the methodology used, particularly in relation to the genotype frequency comparison (χ2=16.75; p<0.001).

Author’s response: I have included this information in the methodology section as follows:

*“.....frequency distributions of Monpa males and females falling under the I/I, I/D, and D/D genotypes were calculated and then compared statistically by using Pearson Chi-square test. Additionally, frequency distributions of –I and –D alleles were calculated and then compared with the help of Pearson Chi-square test.....”*(p 7).

2. **Interpretation of Results**: The findings related to sex differences in anthropometric and physiological variables, as well as the ACE gene polymorphism, are interesting. However, the Discussion could benefit from a deeper analysis of how these results relate to the broader context of genetics and adaptation, especially in the Tawang Monpa population.

Author’s response: I am thankful to the academic editor for this important suggestion. Following this suggestion, I have included a brief description of this information in the Discussion section as follows:

*“………..Evolutionary significance of sex differences in anthropometric and physiological measures is still unclear, as the trend is not comparable across human populations. However, in line with the present study, sex differences in physiological attributes were also reported in previous studies……..”*(p 11).

3. **Figure and Table Presentation**: Please consider improving the presentation of tables and figures, ensuring that they are self-explanatory and align with the text.

Author’s response: I have considered this suggestion and modified the tables as well as both the figures accordingly.

4. **Blood Pressure and ACE Genotype Analysis**: The result that there is no significant difference (p>0.05) among the I/I, I/D, and D/D genotypes in blood pressure levels and other physiological characteristics needs a more comprehensive discussion. It would be insightful to further explore and discuss why these findings are significant and how they contribute to our understanding of ACE gene polymorphism in the population studied.

Author’s response: I agree with this suggestion and so following the advice, I have included additional discussion on this topic in the Discussion section as follows:

“.*........Further, no significant association (p>0.05) between ACE gene polymorphism and physiological characteristics was observed among Monpa, which suggests that ACE gene doesn’t influence blood pressure, lung capacities and/or [Hb] concentration in the population studied...”*(p. 12 & 13).

“.*.....This phenomenon perhaps indicates that ACE gene doesn’t influence arterial blood pressure in the studied population....”* (p. 13).

5. **Language and Formatting**: Finally, there are minor language and formatting issues that need to be addressed to enhance the readability and professionalism of the manuscript.

Author’s response: Following this suggestion, I have taken professional help to edit the manuscript.

**Additional Editor’s Comments:**

Author’s response: I am thankful to the additional editor for the comments. I have incorporated all the suggestions and given point-by-point answers in the above section.
